# International validation of the distal pancreatectomy fistula risk score: evaluation in minimally invasive and open surgery

**DOI:** 10.1007/s00464-025-11872-5

**Published:** 2025-06-20

**Authors:** Philip C. Müller, Suna Erdem, Christoph Kuemmerli, Felix Nickel, O. H. Fiete Gehrisch, Faik G. Uzunoglu, Amelie Hannoschöck, Noa L. E. Aegerter, Caroline Berchtold, Jan Philipp Jonas, Michael C. Frey, Beat Moeckli, Christian Toso, Julia Mühlhäusser, Jörn-Markus Gass, Riccardo Pellegrini, Umberto Cillo, Giovanni Marchegiani, Cristiano Guidetti, Fabrizio Di Benedetto, Anna S. Wenning, Beat Gloor, Kim C. Wagner, Karl J. Oldhafer, Christoph Tschuor, Paul Suno Krohn, Stefan K. Burgdorf, Alberto García-Picazo, Patricia Sánchez-Velázquez, Didier Roulin, John B. Martinie, Thilo Hackert, Beat P. Müller, Adrian T. Billeter

**Affiliations:** 1 Department of Surgery, Clarunis University Digestive Health Care Center, University Hospital Basel, Spitalstrasse 21, 4031 Basel, Switzerland; 2 Department of General, Visceral and Thoracic Surgery, University Medical Center Hamburg-Eppendorf, Hamburg, Germany; 3 Department of Surgery and Transplantation, University Hospital Zurich, Zurich, Switzerland; 4 Department of Surgery, University Hospitals of Geneva, Geneva, Switzerland; 5 Department of General Surgery, Cantonal Hospital of Lucerne, Lucerne, Switzerland; 6 Department of Health Sciences and Medicine, University of Lucerne, 6002 Lucerne, Switzerland; 7 Hepato Pancreato Biliary (HPB) and Liver Transplant Surgery, Department of Surgical, Oncological and Gastroenterological Sciences (DiSCOG), University of Padua, Padua, Italy; 8 Hepato-Pancreato-Biliary Surgery and Liver Transplantation Unit, University of Modena and Reggio Emilia, Modena, Italy; 9 Department of Visceral Surgery and Medicine, Insel, Bern University Hospital, University of Bern, Bern, Switzerland; 10 Department of Surgery, Division of Hepatobiliary and Pancreatic Surgery, Asklepios Hospital Barmbek, Hamburg, Germany; 11 Department of Digestive Diseases, Transplantation and General Surgery Rigshospitalet, Copenhagen University Hospital, Copenhagen, Denmark; 12 Hepato-Biliary and Pancreatic Surgery Unit, Department of Surgery, Hospital del Mar, Pompeu Fabra University, Barcelona, Spain; 13 Division of HPB Surgery, Department of Surgery, Carolinas Medical Center, Charlotte, USA; 14 Department of Visceral Surgery, Lausanne University Hospital and Lausanne University, Lausanne, Switzerland

**Keywords:** Robotic surgery, Pancreatic surgery, Distal pancreatectomy, Outcome research, Complications

## Abstract

**Background:**

Postoperative pancreatic fistula (POPF) remains the most severe complication following distal pancreatectomy (DP). The preoperative distal fistula risk score (D-FRS) was introduced to predict the POPF risk. The aim of this study was to externally validate the D-FRS in an international expert center cohort.

**Methods:**

This international, multicenter, retrospective cohort study included open and minimally invasive DP for benign and malignant lesions performed from 01/2014 until 12/2023 in 12 centres from 6 countries, that each performed more than 50 pancreatectomies annually. The D-FRS was calculated from pancreatic thickness and duct size. Predicted and actual POPF were compared using sensitivity, specificity and area under the curve (AUC).

**Results:**

A total of 778 patients underwent DP of whom 284 (39%) underwent robotic, 278 (38%) open and 165 (23%) laparoscopic DP. The rate of POPF was 32%. The sensitivity, specificity and AUC of the D-FRS for the overall cohort was 32%, 63% and 48% (95% CI 44–51%), respectively. The AUC for open, laparoscopic and robotic DP was 54% (48–60%), 47% (39–55%) and 45% (39–50%), respectively. For neoadjuvant therapy naïve patients the AUC was 52.3%. On multivariate analysis POPF was associated with body mass index (odds ratio 1.04 (95% CI 1.01–1.07)), protective factors were neoadjuvant therapy (OR 0.54 (0.22–0.94)) and the robotic approach (OR 0.64 (0.42–0.97)).

**Conclusions:**

The preoperative D-FRS showed insufficient discrimination to identify patients who develop POPF after DP irrespective of the surgical approach. Novel preoperative POPF risk scores are needed, considering the standard minimally invasive approach and the widespread use of neoadjuvant therapy.

Distal pancreatectomy (DP), with or without splenectomy, is the preferred treatment for benign and malignant tumors located in the body or tail of the pancreas [1]. When performed in specialized, high-volume centers the mortality associated with DP has declined to less than 2% [2]. However, even in benchmark series morbidity following DP remains high, ranging from 29 to 47%, with postoperative pancreatic fistula (POPF) being the most relevant [3, 4]. Despite several efforts to reduce POPF—such as pancreatic stump coverage, perioperative somatostatin analogues, and the omission of intra-abdominal drains—its incidence remains high [5–9]. POPF after DP is associated with re-interventions, prolonged hospital stay, readmissions and mortality [10]. These complications seriously impact the patients’ quality of life and healthcare resources [11].

Following pancreatoduodenectomy (PD) both the original fistula risk score (FRS) and the alternative FRS (a-FRS) were developed to predict POPF [12, 13]. They assess the risk of POPF after PD based on pancreatic texture, duct diameter, intraoperative blood loss, and pathological findings [14, 15].

However, for DP different risk factors have been identified including age, body mass index (BMI), hypoalbuminemia, non-malignant pathology, concomitant splenectomy, and vascular resection [16–19]. Though, pancreas-specific parameters were rarely included until the distal pancreatectomy FRS (D-FRS) was recently developed based on pancreatic duct size and pancreatic thickness [20]. Based on different parameters the D-FRS may be assessed at two time points: as a preoperative score to guide preventive strategies and intraoperatively to aid mitigation efforts.

The objective of this international multicenter study was to validate the preoperative D-FRS for patients undergoing minimally invasive and open DP for both benign and malignant lesions of the pancreatic tail. This external validation aims to assess the score’s accuracy in predicting POPF in a large international patient cohort.

## Methods

### Study design

This study adheres to the STROBE/STROCSS guidelines. Included were high-volume centers performing more than 50 pancreatic resections annually and main surgeons had a personal experience of > 50 DP. The final collaboration involved 12 centers: five in Switzerland (Basel, Bern, Geneva, Lucerne and Zurich), one in Denmark (Copenhagen), two in Italy (Modena and Padova), two in Germany (Hamburg-Eppendorf and Hamburg), one in Spain (Barcelona), and one in the United States (Charlotte). Patients undergoing DP via open, laparoscopic, or robotic approaches between January 1, 2014, and December 31, 2023, were included. The study received approval from the Swiss ethics committee (ID: 2023–02081) and the ethics committees of the individual participating centers.

### Data collection and outcomes

Data were extracted from prospectively maintained institutional databases and stored in a secure, anonymized online data management system. Baseline demographic variables included patient age, sex, BMI, ASA classification, Charlson Comorbidity Index, history of acute or chronic pancreatitis, and prior neoadjuvant therapy. Intraoperative outcomes such as surgical approach (open, laparoscopic, or robotic), type of DP resection, operative time, blood loss, transection technique, pancreatic remnant coverage, and splenectomy status were also recorded [21]. Postoperative complications within 90 days were categorized using the Clavien-Dindo classification; major complications were defined as Clavien-Dindo Grade IIIa or higher [22]. Pancreas specific complications—including POPF [23], post-pancreatectomy hemorrhage (PPH) [24], and delayed gastric emptying (DGE) [25]—were classified according to ISGPS criteria. Textbook outcome (TBO) reflects optimal surgical results after pancreatic surgery and was defined by the absence of POPF, bile leak, PPH, major complications, readmission, and in-hospital mortality [26]. The Pancreatic Surgery Composite Endpoint (PACE) was developed as an alternative measure to assess ideal outcomes following pancreatic surgery. The PACE is positive if one of the following postoperative complications is present: POPF, PPH, reoperation or reintervention. The PACE can be used to predict prolonged hospital stays and perioperative mortality [27].

### Distal pancreatectomy fistula risk score

The preoperative D-FRS was assessed according to its original description by De Pastena et al. [20]: The pancreatic thickness and duct size were assessed using the final preoperative computed tomography (CT) or magnetic resonance imaging (MRI) scan. Measurements were taken at the pancreatic neck, corresponding to the level of the confluence of the spleno-mesenteric vein and were performed by trained surgeons. A specific instruction on how to perform the measurements was handed out to every local principal investigator of the study. Pancreatic thickness was measured in a direct anterior–posterior line to mimic the surgical transection plane, while the pancreatic duct size was measured along its natural course.

### Statistical analysis

Qualitative variables were expressed as frequencies and percentages and quantitative variables as mean and standard deviation (SD), if normally distributed and as median and interquartile range (iqr) otherwise. Comparison of means between groups was done by Student’s *t*-test (pooled *t*-test) or with the nonparametric Mann Whitney *U* test. To compare percentages between the group of patients with and without POPF, a contingency table analysis was used with the chi-square test or Fisher’s exact test when the frequency of cases was low, adding a study of standardized residuals to see the directionality of the associations.

The D-FRS was calculated according to the formula previously published and if the predicted probability of developing a POPF was above 50%, this was considered a POPF and if below or equal to 50%, it was defined as no POPF [23]. Predicted and actual POPF were compared using a matrix to generate sensitivity, specificity and area under the curve (AUC). As the D-FRS was developed in a cohort treated mainly with open DP, a subgroup analysis stratified by surgical approach (open, laparoscopic, robotic) was done. Likewise, the original D-FRS was developed in a cohort with few patients undergoing neoadjuvant treatment. Since neoadjuvant treatment is known to alter pancreatic tissue characteristics to some extent inducing fibrosis and atrophy, it may affect duct size and gland texture [28]. Therefore, a sensitivity analysis was performed in patients without neoadjuvant therapy to assess the performance in this patient group.

To assess associations in this cohort, an adjusted logistic regression model was developed. For the multivariable model, multiple imputation using multivariate imputation by chained equations for a missing at random pattern was performed. Logistic regression on all 5 imputed datasets was run and likelihood ratio statistics were pooled according to the method of Meng and Rubin [29]. Backward variable selection was used with a p-value cut-off of 0.2 and forcing established risk factors for POPF (age, BMI) as well as the surgical approach (open, laparoscopic, robot) into the model. Alternatively, to assess the impact of duct diameter and thickness of the pancreas, those two variables were also forced into the model. Bootstrapping in each imputed dataset and 10 times resampling were performed to assess model performance by the AUC.

## Results

### Patient demographics

A total of 778 patients were included in the study, with a median age of 66 (55–74) years and 50.1% being male. The median BMI was 26 (23–29) kg/m^2^, diabetes was present in 24.8%, and the median Charlson Comorbidity Index was 4 (2–5). The most frequent indications were pancreatic ductal adenocarcinoma (PDAC, 30.9%), neuroendocrine tumors (NET, 22.9%) and intraductal papillary mucinous neoplasia (IPMN, 16.3%). Median pancreatic thickness was 15 (12–18) mm and median pancreatic duct size was 2 (2–3) mm. The characteristics of the entire population are listed in Table 1.

**Table 1:** Patient characteristics

	Total cohort (*n* = 778)	POPF (*n* = 249)	No POPF (*n* = 529)	*P*
Age, years	66 [55–74]	64 [53–72]	67 [56–74]	0.009
Male	390 (50.1)	132 (53.0)	258 (48.8)	0.305
BMI, kg/m^2^	26 [23–29]	26 [24–30]	25 [23–29]	0.011
ASA				0.855
1	31 (4.0)	12 (4.9)	19 (3.6)	
2	368 (47.7)	119 (48.2)	249 (47.5)	
3	353 (45.8)	110 (44.5)	243 (46.4)	
4	19 (2.5)	6 (2.4)	13 (2.5)	
CCI	4 [2–5]	4 [2–5]	4 [2–6]	0.268
Diabetes mellitus	193 (24.8)	57 (22.9)	136 (25.8)	0.439
Acute pancreatitis	68 (11.2)	24 (11.8)	44 (10.9)	0.852
Chronic pancreatitis	83 (13.7)	28 (13.8)	55 (13.6)	1.000
Neoadjuvant therapy	58 (8.1)	15 (6.3)	43 (9.0)	0.262
Pancreatic thickness, mm	15 [12–18]	14 [11–17]	15 [12–18]	0.110
Pancreatic duct size, mm	2 [2, 3]	2 [2, 3]	2 [2, 3]	0.367
Soft pancreas texture	188 (46.4)	67 (58.8)	121 (41.6)	0.003
Histologic diagnosis				0.046
PDAC	236 (30.9)	64 (25.8)	172 (33.4)	
NET	175 (22.9)	58 (23.4)	117 (22.7)	
IPMN	124 (16.3)	43 (17.3)	81 (15.7)	
MCN	40 (5.2)	20 (8.1)	20 (3.9)	
SCN	35 (4.6)	10 (4.0)	25 (4.9)	
Other	153 (19.7)	53 (21.3)	100 (18.9)	
Approach				0.030
Open	278 (38.2)	92 (40.7)	186 (37.1)	
Robotic	284 (39.1)	73 (32.3)	211 (42.1)	
Laparoscopic	165 (22.7)	61 (27.0)	104 (20.8)	
Resection type				0.159
1	559 (78.3)	170 (77.3)	389 (78.7)	
2	59 (8.3)	15 (6.8)	44 (8.9)	
3	86 (12.0)	29 (13.2)	57 (11.5)	
4	10 (1.4)	6 (2.7)	4 (0.8)	
Pancreatic remnant coverage				0.003
Autologous	67 (8.6)	13 (5.2)	54 (10.2)	
Sealant	69 (8.8)	25 (10.0)	44 (8.3)	
Other	19 (2.4)	7 (2.8)	12 (2.3)	
Transection technique				0.001
Stapler	543 (76.4)	161 (72.9)	382 (78.0)	
Knife	57 (8.0)	26 (11.8)	31 (6.3)	
Radiofrequency	64 (9.0)	11 (5.0)	53 (10.8)	
Other	47 (6.0)	23 (9.2)	24 (4.5)	
Blood loss, ml	200 [75–500]	200 [100–500]	150 [50–470]	0.106
Operative time, min	251 [195–310]	260 [204–323]	245 [190–305]	0.098
Conversion rate	87 (13.2)	32 (16.1)	55 (11.9)	0.183
Spleen-preserving	200 (25.7)	136 (25.8)	64 (25.7)	1.000
Splenectomy				0.053
Planned	530 (68.1)	164 (65.8)	366 (69.2)	
Unplanned	47 (6.0)	21 (8.4)	26 (4.9)	
POPF				< 0.001
B	228 (29.4)	228 (91.6)	0 (0)	
C	21 (2.7)	21 (8.4)	0 (0)	
PPH				0.019
B	10 (1.3)	6 (2.4)	4 (0.8)	
C	16 (2.1)	10 (4.0)	6 (1.1)	
DGE				0.068
B	29 (3.7)	14 (5.6)	15 (2.8)	
C	6 (0.8)	4 (1.6)	2 (0.4)	
Reintervention	165 (21.7)	126 (53.4)	39 (7.5)	< 0.001
Reoperation	74 (9.6)	47 (19.0)	27 (5.1)	< 0.001
Highest CD Complication				< 0.001
3a	138 (25.0)	103 (43.8)	35 (11.0)	
3b	58 (10.5)	41 (17.4)	17 (5.3)	
4a	20 (3.6)	8 (3.4)	12 (3.8)	
4b	3 (0.5)	3 (1.3)	0 (0.0)	
5	22 (2.8)	12 (4.8)	10 (1.9)	
Textbook outcome	389 (50.0)	0 (0)	389 (73.5)	< 0.001
Pancreatic surgery composite endpoint	303 (38.9)	249 (100)	54 (10.3)	< 0.001
LOS, days	9 (6 – 14)	8 (6—12)	11 (7 – 20)	< 0.001
Readmission rate	143 (18.6)	102 (41.6)	41 (7.8)	< 0.001

Data are given as *n* (%) and median (IQR)

*BMI* body mass index, *CCI* Charlson Comorbidity Index, *CD* Clavien-Dindo, *DGE* delayed gastric emptying, *LOS* length of stay, *POPF* postoperative pancreatic fistula, *PPH* postpancreatectomy hemorrhage, *PDAC* pancreatic ductal adenocarcinoma, *NET* neuroendocrine tumor, *IPMN* intraductal papillary mucinous neoplasia, *MCN* mucinous cystic neoplasm, *SCN* serous cystic neoplasm, *SPN* solid pseudopapillary neoplasm

### Perioperative outcomes

284 (39.1%) underwent robotic, 278 (38.2%) open and 165 (22.7%) laparoscopic DP. The median operative time was 251 (195 – 310) minutes, and the median blood loss was 200 (75–500) ml. The pancreas was most often transected with a stapler (76.4%), radiofrequency (9.0%) or a knife (8.0%) and pancreatic remnant coverage was performed in a minority of cases (19.8%).

Major complications occurred in 30.1% leading to a reintervention and reoperation rate of 21.2% and 9.6% respectively. The rate of POPF, DGE and PPH was 32.1%, 4.5%, 3.4%. The 90-day mortality rate was 2.8%. The median length of hospital stay was 9 (6–14) days, with a readmission rate of 18.4%.

### Characteristic of patients with POPF

Patients with POPF were younger (64 (53–72) years vs 67 (56–74) years; *p* = 0.009) and had a higher BMI (26 (24–30) kg/m^2^ vs 25 (23–29) kg/m^2^; p = 0.011). Both pancreatic thickness (14 (11–17) mm vs 15 (12–18) mm; *p* = 0.110) and pancreatic duct size (2 (2–3) mm vs 2 (2–3; *p* = 0.367) mm were not significantly different in the two groups. However, soft pancreatic texture was more frequent in the POPF cohort (58.8% vs 41.6%; *p* = 0.003).

### Impact of POPF on perioperative outcomes

There was a significant difference in POPF occurrence between the laparoscopic-, open-, and robotic approach (37.0% vs 33.1% vs 25.7%; *p* = 0.003). In the POPF group the pancreas was less often transected with the stapler (72.9% vs 78.0%) and more frequently with the knife (11.8% vs 6.3%) compared to the no POPF group (both *p* = 0.001). The pancreas remnant was furthermore less frequently covered with autologous material in the POPF group (5.2% vs 10.2%).

Postoperatively, POPF was linked to higher rates of major complications (65.9% vs 13.2%; *p* < 0.001), reinterventions (53.4% vs 7.5%; *p* < 0.001), reoperations (19.0% vs 5.1%; *p* < 0.001) and a higher 90-day mortality rate (4.8% vs 1.9%; *p* = 0.039). Patients with POPF experienced a longer hospital stay (11 (7–20) days vs 8 (6–12) days; *p* < 0.001) and higher readmission rates (41.6% vs 7.8%; *p* < 0.001). PDAC was less common in the POPF group (25.8% vs 33.4%, *p* = 0.046). The comparison of the POPF and no-POPF patients is shown in Table 1.

### Performance evaluation of the D-FRS

The median D-FRS for the entire cohort was 0.23 (0.13–0.36). In the POPF group, the D-FRS was 0.21 (0.13–0.33), while in the no-POPF group it was 0.23 (0.15–0.36). The sensitivity, specificity and AUC of the D-FRS for the overall patient cohort was 32.4%, 62.9% and 47.8%, respectively. In the subgroup analysis the performance of the D-FRS was evaluated for the different surgical approaches. For open DP, the sensitivity, specificity and accuracy were 34.8%, 73.1 and 54.2%, for laparoscopic DP 23.0%, 72.1% and 47.0% and for robotic DP 38.0%, 47.8% and 44.6%. For patients without neoadjuvant therapy the sensitivity, specificity and accuracy were 31.5%, 63.0% and 52.3%.

### Independent risk factors for POPF

A higher BMI (OR 1.04 (1.01–1.07); *p* = 0.017) was associated with POPF in the multivariate analysis. Both neoadjuvant therapy (OR 0.46 (0.22–0.94); *p* = 0.034) and the robotic approach (OR 0.64 (0.42–0.97); *p* = 0.036) resulted in a reduced risk of developing POPF. Conversely, factors such as pancreas thickness and duct diameter were not relevant in the multivariate analysis (Table 2).

**Table 2:** Multivariable logistic regression model

	OR	95% CI	*p* value
(Intercept)	0.45	0.13–1.54	0.203
Age	0.99	0.98–1.00	**0.026**
BMI	1.04	1.01–1.07	**0.017**
Neoadjuvant therapy	0.46	0.22–0.94	**0.034**
Pancreatic thickness	0.97	0.94–1.01	0.097
Pancreatic duct diameter	1.06	0.96–1.16	0.255
Stapler transection	0.76	0.54–1.07	0.119
Operative time	1.00	1.00–1.00	0.057
Approach (compared to open)			
Laparoscopic	1.20	0.79–1.82	0.386
Robotic	0.64	0.42–0.97	**0.036**
Resection type (compared to standard resection)			
With venous resection	0.55	0.28–1.11	0.093
With multivisceral resection	1.16	0.65–2.08	0.615
With arterial resection	4.60	1.13–18.70	**0.033**

Bold numbers represent significant *p* values below 0.05

*BMI* body mass index

## Discussion

In this large international cohort of 778 patients from 12 high-volume pancreatic centers, the preoperative D-FRS demonstrated insufficient discrimination abilities to predict POPF after DP. In a contemporary, mixed patient cohort, where two thirds of patients were operated with a minimally invasive approach, a limited accuracy of the D-FRS was found for both minimally invasive and open DP. The study furthermore confirmed, that POPF after DP remains a highly relevant clinical problem associated with a relevant rate of major complications, re-interventions and a more than two-fold postoperative mortality rate. Importantly, modern treatment concepts including the application of neoadjuvant therapy and widespread use of minimally invasive DP were both shown to be important protective factors for POPF.

In this external validation study, the insufficient accuracy of the D-FRS cohort can be explained by the fact that both pancreatic duct diameter and gland thickness were not risk factors for POPF and not different in patients with and without POPF. This indicates a limited discriminatory ability of the two parameters. However, BMI, neoadjuvant chemotherapy and a robotic approach (the latter being both protective) were associated with POPF in multivariate analysis and in line with previously reported risk factors [30–32]. This is in stark contrast to the original development cohort of 339 patients where pancreatic duct size (OR 1.14 per mm) and pancreatic neck thickness (OR 1.46 per mm) were the only risk factors for POPF, while preoperative chemotherapy and the robotic approach were not considered in this analysis [20]. Especially robotic DP seems to be a protective factor in patients without visceral obesity. In a large propensity matched cohort of 445 patients, the robotic approach resulted in an almost three-times lower POPF rate (10% vs 27%). Interestingly, the learning curve for minimally invasive DP seems to have a limited influence on the POPF rate. In three large multicenter cohorts, the POPF rate remained stable along the competency, proficiency, and mastery phase of minimally invasive DP [33–37].

The patient characteristics of the current study and the original development cohort were comparable regarding BMI, age, ASA classification, duct diameter and gland thickness (median 2 and 15 mm in both cohorts respectively). Of note, in the current cohort the POPF rate was higher (32% vs 23%) and more patients were operated with a minimally invasive approach (58% vs 38%). This may further explain the poor performance of the D-FRS, but reflects the fact that the minimally invasive approach has become the international standard of care in most expert centers [1, 38].

The initiators of the D-FRS validated their score in a nationwide Dutch retrospective cohort of 896 patients, with a malignant indication in 42% of patients, while half of the patients were operated with a minimally invasive approach and 17% developed a POPF. The preoperative D-FRS showed an acceptable AUC of 0.73, similar to the one in the original description [20, 39]. Interestingly, pancreas specific measurements for the development of the D-FRS were performed by experienced radiologist, while in the Dutch validation study and in our own cohort, those evaluations were performed by trained surgeons. On one hand, this might have introduced a certain inter-observer bias, on the other hand, this setting reflects more the “real-life scenario”, where surgeons and not radiologist preoperatively evaluate the risk for POPF. This underlines the necessity of the risk score to be objective, easily assessable and reproducible by different teams. In all three studies, the measurements of the pancreas were performed on the last preoperative CT or MRI at the level of the spleno-mesenteric confluence as illustrated by De Pastena et al. [20]. The pancreatic thickness was measured in a straight anterior–posterior line to simulate the surgical transection plane and the pancreatic duct size in its running course. The different imaging modalities might however further introduce a measurement bias.

While risk factors for POPF after pancreatoduodenectomy are clearly defined and include soft pancreatic texture, small pancreatic duct diameter and high body mass index, the risk factors for DP are less clear and diverse [13, 40]. In a large multinational study of 2′026 DP independent risk factors for POPF included age, obesity, hypoalbuminemia, absence of epidural anesthesia, NET or nonmalignant pathology, vascular resection, concomitant splenectomy and intraoperative drainage placement. However, combining those risk factors led to a model with poor discriminatory power with a c-statistic of 0.654 [16]. The difficult definition of risk factors for POPF after DP may be explained by three considerations: 1. Important risk factors are not yet measured or discovered 2. POPF after DP is an unpredictable stochastic process and 3. Transection techniques and coverage of the remnant significantly impacts POPF, however their applications remain diverse in the published reports.

With regard to unmeasured risk factors, it was suggested that pancreas specific factors like duct size, gland thickness or histological composition are key contributors to POPF risk [41–43]. However, examining the histopathologic features in 102 patients undergoing DP, the amount of steatosis, degree of fibrosis, and pancreatic duct size at the resection margin did not correlate with the formation of POPF. Even dividing the pancreatic parenchyma in “fatty” versus “non-fatty” based on a cutoff of ≥ 10% steatosis showed no correlation with POPF occurrence, suggesting that pancreas texture might not be a key factor in DP [44].

For patients with a high risk for POPF, different surgical mitigation strategies can be applied. Level one evidence from a meta-analysis of 18 randomized controlled trials confirmed that pancreatic remnant coverage decreased the POPF rate after DP by 31%, with reduced rates of mortality, reoperations, and re-interventions consecutively. A POPF reduction was achieved regardless, if autologous or artificial coverage material was applied and can be recommended as standard POPF mitigation technique during DP [8]. However, the rationale of treating the pancreatic stump with different methods to reduce the incidence and burden of a POPF after DP has failed to be proven as key.

In this same direction, two randomized controlled trials and 6 nonrandomized comparative studies support a standard no-drainage policy after DP [45]. The omission of drains in a total of 3′610 patients was not only associated with lower POPF rates, but again with lower major morbidity, less reinterventions and fewer readmissions [9, 46, 47]. While fluid collections are seen in almost every other patient on postoperative imaging, only a minority will become symptomatic and even less require a specific treatment, mostly with an image-guided drainage. Interestingly, surgical drains did not decrease the likelihood of fluid collections, but increased the need for reinterventions, confirming that intraoperatively placed drains dislocate early and often and are therefore not in the right place to adequately drain a POPF [48, 49].

With the limited accuracy of the D-FRS, novel approaches are needed to assess the POPF risk objectively. With the introduction of machine learning and artificial intelligence, an intriguing possibility is texture analysis of medical imaging to define quantitative features unrecognizable by the human eye. These so called radiomic features can uncover valuable parameters or patterns on preoperative imaging for POPF prediction. In seven studies, radiomics of both CT and MRI have shown excellent predictive power for POPF after pancreatoduodenectomy with an AUC between 0.76 and 0.95 [50–52]. While multiple groups already assessed the potential of radiomics to predict POPF after pancreatoduodenectomy, no such study is currently available for DP.

The results of this study should be interpreted in the light of some limitations. An important confounding factor remains the variation in center-specific perioperative management, including intra- and postoperative POPF mitigation strategies such as drainage policy and management, coverage of the pancreatic remnant, and complication management. All of these factors introduce heterogeneity of the assessed outcomes. Second, pancreatic duct size and thickness measurements were not controlled by a second examinator, however these pancreas specific measurements were shown to be reproducible in the Dutch validation study. Finally, the D-FRS was retrospectively applied to the present study cohorts and possible bias intrinsic to the nature of data collections should be disclosed.

In conclusion, this international, multicenter cohort study the preoperative D-FRS showed insufficient discrimination abilities to predict POPF after DP. The accuracy of the D-FRS was limited irrespective of an open- or minimally invasive approach. Novel preoperative POPF assessment strategies are needed, taking into account novel surgical strategies such as the robotic approach, neoadjuvant therapy and the widespread application of a no-drain policy.

## Data Availability

The data that support the findings of this study are available from the corresponding author, [PM], upon reasonable request.
